# Recombination and Its Impact on the Genome of the Haplodiploid Parasitoid Wasp *Nasonia*

**DOI:** 10.1371/journal.pone.0008597

**Published:** 2010-01-19

**Authors:** Oliver Niehuis, Joshua D. Gibson, Michael S. Rosenberg, Bart A. Pannebakker, Tosca Koevoets, Andrea K. Judson, Christopher A. Desjardins, Kathleen Kennedy, David Duggan, Leo W. Beukeboom, Louis van de Zande, David M. Shuker, John H. Werren, Jürgen Gadau

**Affiliations:** 1 School of Life Sciences, Arizona State University, Tempe, Arizona, United States of America; 2 Center for Evolutionary Functional Genomics, The Biodesign Institute, Arizona State University, Tempe, Arizona, United States of America; 3 Institute of Evolutionary Biology, University of Edinburgh, Edinburgh, United Kingdom; 4 Centre for Ecological and Evolutionary Studies, Evolutionary Genetics, University of Groningen, Haren, AA, The Netherlands; 5 Department of Biology, University of Rochester, Rochester, New York, United States of America; 6 Translational Genomics Research Institute, Phoenix, Arizona, United States of America; 7 School of Biology, University of St Andrews, St Andrews, United Kingdom; Landcare Research, New Zealand

## Abstract

Homologous meiotic recombination occurs in most sexually reproducing organisms, yet its evolutionary advantages are elusive. Previous research explored recombination in the honeybee, a eusocial hymenopteran with an exceptionally high genome-wide recombination rate. A comparable study in a non-social member of the Hymenoptera that would disentangle the impact of sociality from Hymenoptera-specific features such as haplodiploidy on the evolution of the high genome-wide recombination rate in social Hymenoptera is missing. Utilizing single-nucleotide polymorphisms (SNPs) between two *Nasonia* parasitoid wasp genomes, we developed a SNP genotyping microarray to infer a high-density linkage map for *Nasonia*. The map comprises 1,255 markers with an average distance of 0.3 cM. The mapped markers enabled us to arrange 265 scaffolds of the *Nasonia* genome assembly 1.0 on the linkage map, representing 63.6% of the assembled *N. vitripennis* genome. We estimated a genome-wide recombination rate of 1.4–1.5 cM/Mb for *Nasonia*, which is less than one tenth of the rate reported for the honeybee. The local recombination rate in *Nasonia* is positively correlated with the distance to the center of the linkage groups, GC content, and the proportion of simple repeats. In contrast to the honeybee genome, gene density in the parasitoid wasp genome is positively associated with the recombination rate; regions of low recombination are characterized by fewer genes with larger introns and by a greater distance between genes. Finally, we found that genes in regions of the genome with a low recombination frequency tend to have a higher ratio of non-synonymous to synonymous substitutions, likely due to the accumulation of slightly deleterious non-synonymous substitutions. These findings are consistent with the hypothesis that recombination reduces interference between linked sites and thereby facilitates adaptive evolution and the purging of deleterious mutations. Our results imply that the genomes of haplodiploid and of diploid higher eukaryotes do not differ systematically in their recombination rates and associated parameters.

## Introduction

Homologous meiotic recombination in eukaryotes is a process by which genetic material is exchanged between homologous chromosomes [1]–[3]. It occurs in almost all sexually reproducing organisms and results in the reshuffling of the genetic material of corresponding pairs of parental chromosomes [4]. As a consequence, the offspring of sexually reproducing organisms carry genotype combinations that are not found in either of their parents [1]–[3]. The evolutionary advantages of homologous meiotic recombination and recombination in general are not immediately apparent. In fact, recombination seemingly contradicts the expectations of Darwinian natural selection, since genotype combinations that have been proven successful in the current environment by natural selection are actively broken up [5]. This is one of the aspects of the “paradox of sex”.

Evolutionary biologists have put forward various hypotheses to explain the evolutionary advantages of recombination [4]–[6]. One of them states that homologous meiotic recombination increases the efficiency of natural selection by alleviating interference between linked sites, thereby facilitating adaptive evolution and the purging of deleterious mutations [7]–[11]. Given that most amino acid changes are slightly deleterious [12] and assuming that most genes in the genome are relatively conserved, one would expect that regions of the genome with a low recombination rate, when compared to those with a high rate, would show i) a higher ratio of non-synonymous (amino acid replacing) to synonymous (silent) substitutions (*ω*) and ii) a higher degree of protein divergence (*d*_A_) due, in both cases, to the reduced effectiveness of selection against slightly deleterious mutations. In addition, differences in the recombination rate should be reflected in the structure of the genome. Functional genomic elements, for example, are expected to be found at higher density in genome regions with a high recombination rate where fewer slightly deleterious mutations accumulate than in areas of low recombination [13]. However, few studies have actually tested these predictions (e.g., [14]–[20]).

Beye et al. [21] analyzed chromosome, sequence, and gene parameters and their association with the recombination rate in the social honeybee, a taxon with a genome-wide recombination rate approximately ten times higher than any other higher Eukaryote studied so far [21]–[23]. However, honeybees are both haplodiploid and eusocial, factors that could both influence genome evolution. A comparable study on the evolution of the genome-wide recombination rate in a non-social Hymenoptera species that would disentangle the impact of eusociality from Hymenoptera-specific features such as haplodiploidy is missing [24], [25]. With the recent sequencing of the *Nasonia* parasitoid wasp genomes [26], we now have the resources to study recombination in the genome of another haplodiploid hymenopteran in similar detail and to contrast the results with those from the eusocial honeybee.

In the present paper, we provide estimates of recombination rates in *Nasonia*, study parameters that could be correlated with the recombination rate, and assess the impact of recombination on the efficiency of natural selection. First, we infer a high-density linkage map for *Nasonia* by analyzing SNP microarray genotype data of recombinant F_2_ hybrids of *N. vitripennis* and *N. giraulti*. We use the map information to order and orient the sequence scaffolds ( = arranged contiguous DNA sequences with interior gaps of known size) of the *N. vitripennis* assembly 1.0 and to estimate recombination rates along all five chromosomes of *Nasonia*. While these estimates are derived from recombination events in an interspecific cross, a separate study that compared intra- and interspecific recombination frequencies in 33 genomic regions distributed across the entire genome indicated that they are likely representative of the intraspecific recombination rates in all three *Nasonia* species (Leo Beukeboom, unpublished; data available upon request). Next, we analyze chromosome, sequence, and gene parameters in the *N. vitripennis* genome and study their association with the recombination rate. Finally, we examine the amino acid divergence and the ratio between synonymous and non-synonymous substitutions per site between *N. vitripennis* and *N. giraulti* or *N. longicornis*, respectively, to assess the impact of recombination on the efficiency of selection in the *Nasonia* parasitoid wasp genome.

## Results

### Linkage Map Reconstruction

We studied a total of 1,645 markers, of which 310 were excluded from the linkage analysis on the grounds that ≥10% of their genotypes were missing and/or one of the two parental genotypes occurred at an improbable frequency (≥70%; Table S1). We removed an additional 77 markers during linkage map reconstruction because they disturbed marker order stability and/or showed genotype combinations that could only be explained by two unlikely simultaneous recombination events in immediate spatial proximity or by mutation (Table S2). The remaining 1,258 markers clustered into seven major groups. Chromosome anchored markers ([27], [28]; Table S3) revealed that five of the seven groups corresponded with the five chromosomes (Chr 1–5) of *Nasonia* and that these five groups comprised 99.9% of all analyzed markers. The remaining two groups included only one and two markers, respectively. We consequently regarded these two groups as questionable and omitted them in the subsequent analyses (Table S4).

**Figure 1:**
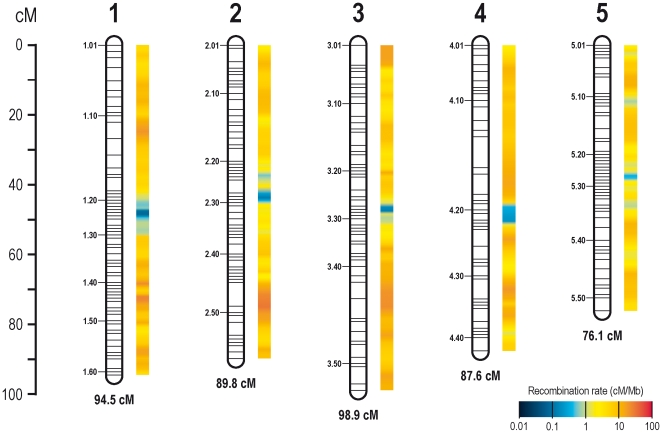
High-density linkage map and estimated recombination rates of the *Nasonia* genome. High-density linkage map of the *Nasonia* parasitoid wasp genome inferred from segregation data of 1,255 markers in a *N. vitripennis* (♀)×*N. giraulti* (♂) F_2_ hybrid population of 112 male embryos. Horizontal bars (1.01–5.51) represent clusters of markers with no recombination among them (see Table S5). The average distance between the 1,255 markers and 265 marker clusters was 0.3 cM and 1.7 cM, respectively. The heat map next to the linkage map depicts the estimated local recombination rate (cM/Mb) in the corresponding region of parasitoid wasp genome. The local recombination rate was approximated for a total of 259 overlapping windows of two consecutive marker clusters each and an average width of 3.4 cM (Table S7).

Our final *Nasonia* linkage map consisted of 1,255 markers on five linkage groups corresponding to the five chromosomes of *Nasonia* and spanned 446.9 cM (Table S5; Fig. 1). The markers mapped in 264 marker clusters (i.e., groups of markers in which the markers of a group did not recombine; Fig. 1: 1.01–5.51) with an average distance between the clusters of 1.7 cM (min/max: 0.9/8.8 cM). The average distance between individual markers was 0.3 cM. The average frequency of the *N. vitripennis* genotype for markers on the linkage map was 49.5% (min/max: 38.8%/61.2%). We estimated an average number of 1.8 chiasmata per chromosome per meiosis and a total of 499 recombination events to explain the observed distribution of genotypes in the 112 studied F_2_ hybrid males (Table S6). In 113 instances, we found two simultaneous recombination events on a chromosome, and in one instance, we counted three events. The average distance between two recombination events, when observed on a single chromosome, was 46.2 cM (min/max: 6.2/91.8 cM). The above results are summarized in Table 1, which also provides chromosome specific information.

**Table 1:** Characteristics of the mapping population, linkage map, and selected sequence and gene parameters.

	Chromosome					Total
	1	2	3	4	5	
**Number of analyzed male F_2_ hybrid embryos**	112	112	112	112	112	112
**Observed number of recombination events**	106	100	110	99	84	499
**Average number of recombination events per chromosome**	0.95	0.89	0.98	0.88	0.75	0.89
**Observed instances of two/three recombination events per chromosome**	27/0	18/0	28/1	21/0	19/0	113/1
**Average distance between two recombination events (cM)**	49.4	49.4	50.3*	39.7	39.7	46.2
**Min/Max distance between two recombination events (cM)**	14.1/86.3	23.5/80.9	15.5/91.8*	6.2/65.6	17.8/55.5	6.2/91.8
**Number of mapped markers**	372	207	188	224	264	1255
**Average genetic distance between markers (cM)**	0.3	0.4	0.5	0.4	0.3	0.4
**Number of marker clusters**	61	58	53	41	51	264
**Average distance between marker clusters (cM)**	1.6	1.6	1.9	2.2	1.5	1.7
**Min/Max distance between marker clusters (cM)**	0.9/4.8	0.9/6.7	0.9/5.7	0.9/8.7	0.9/4.7	0.9/8.8
**Average ** ***N. vitripennis*** ** genotype frequency (%)**	49.2	52.5	47.5	55.0	44.4	49.5
**Min/Max ** ***N. vitripennis*** ** genotype frequency (%)**	42.0/53.3	45.1/56.3	44.3/60.7	47.3/61.2	39.3/55.4	39.3/61.2
**Genetic length (cM)**	94.5	89.8	98.9	87.6	76.1	446.9
**Mapped scaffolds**	91	48	46	61	31	265
**Mapped scaffolds (orientated)** **	11	10	9	5	7	39
**Physical length (Mb)**	48.3	39.5	34.0	35.8	30.0	187.6
**GC content (%)**	42.2	43.6	43.2	42.8	43.4	43.0
**Simple repeat content (nt/Kb)**	17.0	20.3	19.2	18.7	18.4	18.6
**Average gene density (genes per Mb)**	59.2	58.7	49.7	59.3	66.9	58.6
**Average gene distance (Kb)**	10.8	10.6	14.9	9.2	9.9	11.0
**Average protein size (aa)**	574.6	578.8	595.2	587.2	588.6	583.8
**Average number of exons per gene**	6.4	6.6	6.8	6.7	6.8	6.7
**Average exon size (nt)**	270.9	261.3	264.1	262.2	259.0	264.1
**Average intron size (nt)**	1324.3	1173.0	1311.3	1265.8	1079.5	1240.7
**Average ** ***d*** **_A_ (** ***N. vitripennis*** **/** ***N. giraulti*** **)** ***	0.019	0.015	0.017	0.018	0.016	0.017
**Average ** ***d*** **_A_ (** ***N. vitripennis*** **/** ***N. longicornis*** **)** ***	0.018	0.014	0.016	0.017	0.016	0.017
**Average ** ***ω*** ** (** ***N. vitripennis*** **/** ***N. giraulti*** **)** ****	0.282	0.236	0.267	0.262	0.258	0.262
**Average ** ***ω*** ** (** ***N. vitripennis*** **/** ***N. longicornis*** **)** ****	0.273	0.251	0.252	0.259	0.264	0.261

Characteristics of the studied mapping population of recombinant F_2_ hybrids of *Nasonia vitripennis* and *Nasonia giraulti*, the high-density *Nasonia* linkage map derived from it, and selected sequence and gene parameters of the mapped sequence scaffolds of the *Nasonia* genome assembly 1.0.

* The single instance of three recombination events per chromosome is excluded from the calculation.

** For misaligned scaffolds, we included them if at least one fragment was orientated.

*** *d*_A_ = amino acid divergence.

**** *ω = *ratio of non-synonymous (amino acid replacing) to synonymous (silent) substitution rates.

### Scaffold Mapping

The segregation data of the markers allowed us to map 265 sequence scaffolds of the *Nasonia* genome assembly 1.0 (Table 1; Table S5). For 39 of them, the segregation data also enabled us to orient them on the chromosomes (Table S5). The mapping data indicated that at least twelve scaffolds of the *Nasonia* genome assembly 1.0 are misassembled. For example, markers of scaffold 1 mapped to Chr 1 (1.29–1.58) and Chr 5 (5.19–5.26), suggesting an erroneous concatenation of sequences between the nucleotides 5,823,787 and 5,831,911. The fragment of scaffold 1 on Chr 5 is likely misassembled as well since markers of scaffold 1664 and scaffold 4559 mapped to Chr 5 between those of scaffold 1. The currently known instances of misassembled scaffolds of the *Nasonia* genome assembly 1.0 are summarized in Table 2.

**Table 2:** Misassembled sequence scaffolds.

Scaf	Chromosome				
	1	2	3	4	5
**1**	**1.29–1.58**: 1–5,823,787				**5.19–5.21**: 5,831,911–6,693,166 **5.22**: 7,059,870–7,169,619 **5.24–5.26**: 8,284,408–9,425,349
**5**	**1.25**: 1–33,395	**2.25–2.28**: 135,295–4,548,788			
**6**			**3.16–3.19**: 4,401,492–2,651,642 **3.21–3.26**: 2,502,175–1		
**7**	**1.59–1.61**: 429,779–1				**5.12–5.19**: 4,040,552–710,069
**8**		**2.02–2.04**: 1–907,155	**3.30–3.31**: 3,676,232–960,353		
**17**	**1.24**: 2,656,364–2,710,507		**3.39–3.46**: 669,157–2,316,969	**4.20**: 1–421,253	
**21**	**1.19**: 1–245,801	**2.42–2.49**: 2,981,920–696,328			
**24**	**1.18**: 2,056,045–2,507,974	**2.50–2.58**: 1,831,414–1			
**39**	**1.23–1.24**: 177,399–1,144,834 **1.27**: 1–9,220				
**69**	**1.24**: 1–455,644			**4.22**: 695,384–797,218	
**163**	**1.24**: 378,363–381,988				**5.28**: 1–185,791
**429**			**3.27**: 1–44,229		**5.27**: 68,222–106,947

Misassembled sequence scaffolds of the *Nasonia* genome assembly 1.0.

For each misassembled sequence scaffold ( = Scaf; rows), the table shows the chromosome (columns) and position (bold coordinates, see Figure 1 and Table S5) where a scaffold fragment (non-bold coordinates) mapped. For more information about the position of the markers that had been used to derive the estimates, see Table S5.

### Genome Coverage

The *Nasonia* genome assembly 1.0 consists of 6,181 scaffold sequences with a total of 295.1 Mb [26]. Only 4.3% of these scaffolds are mapped on our linkage map. However, since most of the mapped markers lie on comparatively large scaffolds–for example, all 46 scaffolds larger than 1 Mb are mapped–63.6% (187.6 Mb) of the assembled genome (295.1 Mb) and 60.1% of the total estimated genome size of *N. vitripennis* (312 Mb) are represented by the scaffolds on the present linkage map (Table 1).

### Recombination Rate Estimates

Assuming that the inferred *Nasonia* linkage map is saturated (i.e., the mapping of additional scaffolds would not lead to its expansion) and that the physical size estimate of the *N. vitripennis* genome (312 Mb) is accurate, we estimated a genome wide recombination rate of 1.4 cM/Mb. However, almost all analyzed markers were developed by studying the sequences of the *Nasonia* genome assembly 1.0. Hence, our linkage map might only reflect the fraction of the *Nasonia* genome that has actually been sequenced. We therefore performed a second estimate of the genome-wide recombination rate by dividing the length of the genetic map by the total length of all scaffold sequences of the *Nasonia* genome assembly 1.0 (295.1 Mb; see above) and obtained a marginally higher value: 1.5 cM/Mb.

**Figure 2:**
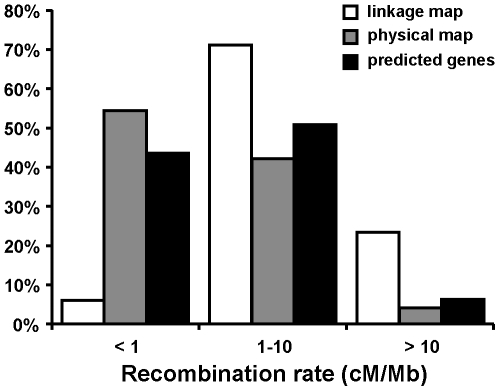
Recombination rates in the linkage map, physical map, and predicted genes. Estimated percentages of the linkage map, the physical map, and of predicted genes of the *Nasonia vitripennis* nuclear genome with low (<1 cM/Mb), medium (1–10 cM/Mb), and high (>10 cM/Mb) recombination rates.

The local recombination rate in *Nasonia* was approximated for a total of 259 overlapping windows of two consecutive marker clusters each and an average width of 3.4 cM (min/max: 1.4/10.7 cM; Table S7). The estimates suggest that the local recombination rate in the *Nasonia* genome varies by two orders of magnitude and lies between 0.08 cM/Mb and 29.6 cM/Mb (Figure 1; Table S7). The bulk (94%) of the *Nasonia* linkage map covers genome regions with an estimated local recombination rate of 1–10 cM/Mb or higher (Figures 1 and 2). The remaining 6% of the map represent regions with an estimated local recombination rate<1 cM/Mb. However, these 6% encompass 54% of the mapped physical genome (Figure 2).

### Association of Chromosome, Sequence, and Gene Parameters with the Local Recombination Rate

Using 125 non-overlapping windows of two consecutive marker clusters each, we assessed the association of the local recombination rate in *Nasonia* with 14 different chromosome, sequence, and gene parameters (average window-width: 3.4 cM; min/max: 1.8/10.7 cM; Table 3; Table S8). For this purpose, we used Dutilleul's modified *t* test [29], which corrects for spatial autocorrelation (since variables sampled spatially close to one another are not necessarily independent from each other). We found that the recombination rate is positively correlated with the physical distance to the center of the linkage groups as well as with GC and simple repeat content (Table 3). In addition, we found a strong correlation of the recombination rate with gene content. In fact, the majority of mapped genes (57%) lay in regions of the mapped genome with an estimated recombination rate of ≥1 cM/Mb (Figure 2). No correlation was found between the recombination rate and the size of proteins, the length of exons or the number of exons per gene (Table 3). The fact that regions of the genome with a high recombination rate tend to have more genes is explained by a strong negative association of the recombination rate and the distance between genes and the size of their introns (Table 3). Finally, we detected an overall negative trend of *d*_A_ (amino acid distance) and *ω* (ratio of non-synonymous to synonymous substitution rates) with the recombination rate, but the only significant association after Bonferroni correction for multiple testing was that of the recombination rate with *ω* when comparing *N. vitripennis* exon sequences with that of *N. longicornis* (Table 3). Average values of the 14 studied parameters are similar over all five chromosomes (Table 1).

**Table 3:** Association of sequence and gene parameters with the local recombination rate.

Parameter	Correlation	Effective sample size	*p*
Physical distance from center of linkage group	0.501	116.8	<0.00001^**^
GC content	0.282	119.1	0.00188^*^
Simple repeat content	0.302	106.5	0.00163^*^
Gene content	0.349	124.6	0.00007^**^
Gene distance	−0.429	121.9	<0.00001^**^
Protein size	0.140	142.5	0.09640
Number of exons per gene	0.044	137.3	0.60965
Exon size	0.098	118.6	0.29026
Intron size	−0.467	122.3	<0.00001^**^
*d*_A_ (*N. vitripennis*/*N. giraulti*)^a^	−0.199	55.8	0.14239
*d*_A_ (*N. vitripennis*/*N. longicornis*)^a^	−0.221	89.2	0.03753
*ω* (*N. vitripennis*/*N. giraulti*)^b^	−0.302	47.9	0.03695
*ω* (*N. vitripennis*/*N. longicornis*)^b^	−0.317	89.6	0.00236^*^

Sequence and gene parameters tested for their association with the local recombination rate in the *Nasonia vitripennis* parasitoid wasp genome.

The analyses were based on data from 125 non-overlapping windows of two consecutive marker clusters each and an average width of 3.4 cM. To account for spatial autocorrelation of the tested parameters, we applied Dutilleul's modified *t* test [29] using ranked data. One and two asterisks, respectively, indicate significant (*α* = 0.05) and highly significant (*α* = 0.01) correlations after Bonferroni correction for 13 simultaneous tests.

a *d*_A_ = amino acid divergence.

b *ω = *ratio of non-synonymous (amino acid replacing) to synonymous (silent) substitution rates.

## Discussion

### Linkage Map Reconstruction

Using genotype data from a SNP microarray, we inferred the first high-density linkage map for *Nasonia* with a total of 1,255 markers on five linkage groups corresponding to the five metacentric to submetacentric chromosomes of *Nasonia* [30]. The small average distance (0.3 cM) between the markers on the five chromosomes suggests that the *Nasonia* linkage map is highly saturated and not likely to expand by the mapping of additional scaffolds. However, when comparing our map with those from prior studies on *Nasonia* that all included less than 100 molecular markers [27], [28], [31]–[33], it is not particularly long (446.9 cM). In fact, the linkage map inferred here is 41.5% shorter than the only other available genome map for *Nasonia* that includes more than 90 markers (764.5 cM; [31]). Does this indicate that we are nevertheless missing parts of the *Nasonia* genome that other maps covered? A closer inspection shows that this concern is most likely unfounded. Rütten et al. [27] reported sequence-tagged site (STS) and microsatellite (MS) markers that they studied together with random amplified polymorphic DNA (RAPD) in *Nasonia*. The size of their linkage map, including RAPD, STS, and MS markers, lies in the same size range as the map published by Gadau et al. [31], which is also based on RAPD markers [34]. When searching the most distant STS and MS markers of each linkage group against the *N. vitripennis* genome, we found that all of them lie in genome regions that are covered by our high-density linkage map. The size differences between the *Nasonia* linkage maps thus appear to have other causes.

The most likely explanation for the discrepancies in the size of the *Nasonia* linkage maps is the differential quality of the genotype data on which they are based and the use of different mapping functions to adjust for undetected recombination events. Almost all linkage maps for *Nasonia* published so far have relied on RAPD or amplified fragment length polymorphic (AFLP) markers [34]. Both types of markers are known to be sensitive to changes in the reaction conditions and hence to frequently miscall genotypes, while in tests the SNP genotyping assay employed here consistently assigned the majority of species-specific genotypes correctly when studying pure strain individuals of *N. vitripennis* and *N. giraulti* (J. D. Gibson, unpubl. results). In particular, we were able to unambiguously identify failed reactions since the SNP genotyping assay allowed us to detect the two competing alleles simultaneously. However, our workflow included additional steps during which we assessed the plausibility of the inferred genotype data. For example, prior analysis of randomly distributed markers in the mapping population of F_2_ hybrid embryos found neither of the two parental genotypes at a frequency >57% [33]. We consequently considered markers showing a genotype at a frequency ≥70% as doubtful and removed them from the dataset. Could our action have led to excluding regions of the genome with an unexpectedly high marker transmission ratio distortion (MTRD)? If regions in the genome of the F_2_ hybrid embryos had a MTRD≥70%, we would expect to find linked markers showing genotype frequencies between 60% and 69%. However, the highest frequency of a genotype seen in our dataset was 60.7%. If the corresponding marker was linked to a region with a MTRD≥70%, it would mean that we had been very unfortunate in not having mapped any additional markers with a MTRD between 61% and 70%. This appears unlikely given the high density of markers on our linkage map.

A second criterion that we applied after an initial ordering of all markers to identify genotypes likely to be false was the occurrence of genotype distributions along the linkage groups of individual recombinant F_2_ hybrids that could only be explained by two simultaneous recombination events in close spatial proximity or by mutation. Specifically, we considered the genotype of a marker that differed from the genotypes of immediately adjacent markers left and right of it as highly improbable due to chiasma interference [35]–[37], and removed the markers with the deviant genotype(s) from the dataset. Could this procedure have caused us to underestimate recombination frequencies? We think this is unlikely to be the case. After having removed all markers with suspect genotypes (77), we counted a total of 113 unambiguous instances of two simultaneous recombination events on a chromosome. The average estimated distance between two recombination events was 46.2 cM and the smallest observed distance was 6.2 cM. These numbers stand in stark contrast to the 482 cases where we observed a single marker with (a) deviant genotype(s) that would have required one to assume two simultaneous recombination events in its immediate vicinity. While we cannot rule out the possibility that we might have discarded a small number of markers whose genotypes provided evidence for two recombination events in close spatial proximity, their impact on the total size estimate of the *Nasonia* linkage map would most likely have been small. In contrast, the consideration of more than 450 false genotypes would have led us to dramatically overestimate recombination in the *Nasonia* genome. Overall, we think that the applied measures to detect and exclude unreliable genotype data significantly improved the accuracy of the *Nasonia* linkage map and that our map reflects recombination frequencies in the *Nasonia* genome far better than previous attempts.

A third and important difference between the current study and that by Gadau et al. [31] is that mapping here was performed on embryos whereas the previous study was performed on adult males. Significant post hatching mortality is known for F_2_ hybrid males between *N. vitripennis* and *N. giraulti* [33], [38] and can result in pseudolinkage effects [39]–[44], an apparent linkage between regions due to epistatic interactions causing mortality which could distort map distances. That said, a comparison of recombination frequencies in intraspecific versus interspecific mapping populations of *Nasonia* by Leo Beukeboom (unpublished; data available upon request) suggests that pseudolinkage may only have a minor impact, if at all, on map distances in *Nasonia*.

### Genome-Wide Recombination Rate

Depending on whether we take the estimated total physical size [45] or the size of the assembled genome of *N. vitripennis* [26] as a basis for the calculation, we estimated the genome-wide recombination frequency in *Nasonia* to be 1.4–1.5 cM/Mb. This is considerably lower than that of the honeybee (19 cM/Mb), the only other Hymenoptera taxon for which an accurate high-density linkage map and a sequenced genome are currently available. Could the genome-wide recombination rate that we estimated for *Nasonia* be so low because we analyzed recombination in an interspecific and not an intraspecific cross? We think this is not the case. Leo Beukeboom studied recombination in intra- and interspecific mapping populations of *N. vitripennis* and *N giraulti* by analyzing 33 genomic segments with an average width of 11 cM (intraspecific cross) distributed across the entire genome (unpublished; data available upon request). He found that recombination frequencies are only marginally (1.8%) higher in the intraspecific cross than in the interspecific cross. This difference thus cannot account for the substantial discrepancy that we see in the genome-wide recombination rate between the parasitoid wasp and the honeybee.

The genome-wide recombination frequency in *Nasonia* lies within the range found in other insect species for which high-density linkage maps based on reliable genotype data exist (e.g., *Drosophila* species [46]; *Bombyx* [47]). Previous studies proposed that the haplodiploid Hymenoptera could have a higher genome-wide recombination rate to compensate for the fact that meiosis occurs only during oogenesis and not during spermatogenesis, because their males are typically haploid [48]. Our data do not support this hypothesis. In fact, we suspect that the slightly higher genome-wide recombination rates for parasitoid Hymenoptera reported by Wilfert et al. [25] are an artifact of inaccurate genetic size estimates. Our estimate of the genome-wide recombination rate in *Nasonia* are, for example, around 40% lower than the estimate (2.5 cM/Mb) referred to by Wilfert et al. [25], which was based on the RAPD marker linkage map for *Nasonia* published by Gadau et al. [31]. Since the three other genetic size estimates for parasitoid wasp genomes considered by Wilfert et al. [25] in their meta-analysis also relied on genotype data of RAPD markers [49]–[51], they could be inflated as well.

Using the data from our high-density linkage map, we estimated an average number of 1.8 chiasmata per chromosome per meiosis for *Nasonia*. This value is only marginally higher than the average number (approximately 1.6) found across a wide range of protist, plant, and animal taxa [5], [52]. It is generally assumed that the relatively uniform number of chiasmata per chromosome per meiosis across taxa is related to the stabilizing function of chiasmata in the pairing of homologous chromosomes during meiosis [53], [54]. However, this mechanistic explanation does not seem to hold for the honeybee, for which an average number of 5.7 chiasmata per chromosome per meiosis has been reported [21]. The substantial difference in the frequency of chiasmata per chromosome per meiosis between *Apis* and *Nasonia* thus raises the question of whether it is caused by a chromosome-wide elevation of the recombination rates or by an increased proportion of genome regions with a high recombination frequency in the honeybee.

### Estimation of Local Recombination Rates and Associated Parameters

In order to infer recombination rates along chromosomes, we estimated the physical size of two consecutive marker clusters (i.e., two consecutive groups of markers in which the markers within a group did not recombine) and divided it by their estimated genetic size (see methods section for further details). The use of pairs of consecutive marker clusters allowed us to obtain upper limit estimates of the recombination rates for genome regions with low recombination frequency and/or discontinuous genome sequence data. Since we calculated the recombination rate for intervals of approximately 3.4 cM–the average estimated size of two consecutive marker clusters on our linkage map–we ensured that a minimum and an approximately equal number of recombination events were included in each calculation.

Traditional approaches infer local recombination rates by calculating the ratio of genetic to physical distance between pairs of markers mapping on the same continuous genome sequence. These ratios are positioned along the chromosomes and a polynomial function is fitted to the data in order to account for local variation in the rates [13]. Despite its widespread use and popularity, this approach has disadvantages: the fitting of a polynomial function across a chromosome artificially increases spatial autocorrelation of recombination rate estimates, which renders most statistical analyses using multiple recombination rate estimates derived from this function inappropriate. The approach is also difficult to apply in regions of the genome with low recombination frequency, unless a huge number of recombinant individuals are screened, since recombination data have to be obtained from continuous genome sequences.

The approach used here to estimate local recombination frequencies tries to cope with both problems mentioned in the previous paragraph. However, it has its own drawbacks as well. Most notably, recombination rates can only be estimated for windows of approximately constant genetic width, but not for windows of constant physical width. The physical size of windows with the same genetic width can consequently differ considerably. There are two main problems associated with different window widths. First, the uncertainty (variance) of the estimates may scale with window width. Second, it is difficult to compare data not estimated at the same physical scale. The first problem is particularly difficult to resolve, since choosing a window of constant genetic width might control for variation in the accuracy of the recombination rate estimates, but this means that the variances associated with sequence and gene parameter estimates may vary across the different physical widths. Likewise, choosing a window of constant physical width might control for the size of the analyzed sequence, but adds a possible bias to the estimates of the recombination rate, unless one dynamically adapts locally the number of analyzed recombinants. Even then, however, the accuracy of sequence and gene parameters that vary significantly within the genome might still differ between windows. Given this trade-off between parameter estimates associated with physical versus genetic width, we think that the decision to employ a window-width that provides reasonably robust estimates across the entire genome, considering both the genetic and the physical dimension, is likely to be the most productive.

### Local Recombination Rates

We found that the recombination rate along the chromosomes varies in *Nasonia* by two orders of magnitude and lies between 0.08 cM/Mb and 29.6 cM/Mb, when estimating the recombination frequencies for windows with a genetic size of approximately 3.4 cM. The variation in the recombination rate for the honeybee genome reported by Beye et al. [21] is considerably larger (0–143 cM/Mb). However, their estimates are for windows with a constant physical width of 125 Kb. A direct comparison between the estimates for the parasitoid wasp and the eusocial honeybee is therefore not possible. More informative are the calculated relative proportions of the *Apis* and of the *Nasonia* genome with low and high recombination frequencies. We found that 54% of the mapped physical genome of the parasitoid wasp has estimated recombination rates<1 cM/Mb (Figure 2). When looking at the entire genome, this estimated fraction is likely to be conservative since we assume that the majority of unmapped scaffold sequences (107.5 Mb) will fall in regions with low recombination. This result contrasts with the much smaller fraction of 9% of the honeybee genome with estimated recombination rates≤5 cM/Mb [21]. Using the same benchmark, we calculate a fraction of over 81% for the parasitoid wasp. Beye et al. [21] further found 12% of the studied honeybee genome sequences (30.7% of the sequenced genome) to have recombination rates>50 cM/Mb. Comparable rates have not been observed in any region of the *Nasonia* genome.

As with the genome-wide recombination estimates, we have to ask whether the discrepancy in the recombination rate estimates for the honeybee and the parasitoid wasp could be due to the fact that we inferred recombination from an interspecific cross and not an intraspecific cross. A study by Leo Beukeboom (unpublished; data available upon request) showed that the difference in the genome-wide recombination rate between inter- and intraspecific mapping populations of *N. vitripennis* and *N giraulti* is almost negligible. This does not mean, however, that the recombination frequency could not differ substantially at a more local scale. While we do not yet have the definitive data to address this caveat, we have reason to believe that at the scale at which we studied local recombination frequencies (approximately 3.4 cM intervals), these differences are likely to be small. Our data suggest that the recombination rate in the *Nasonia* genome varies by at least two orders of magnitude and that adjacent genome regions tend to have similar rates (Figure 1). When comparing recombination frequencies for 33 genomic segments from an intraspecific *N. vitripennis* cross, analyzed by Leo Beukeboom (unpublished; data available upon request), with those from our interspecific *N. vitripennis*×*N. giraulti* cross, we find no single pair of values differing by more than a factor of 3.5 (average 1.4). We therefore think that the striking pattern of local recombination rate variation observed in the *Nasonia* genome would not have been different if we had studied an intraspecific cross.

Overall, whilst the differences in the analyses (e.g., constant genetic vs. constant physical window-width) and in the proportion of the genome (i.e., 63.6% vs. 30.7%) analyzed to identify regions of low- or high recombination in the parasitoid wasp and in the honeybee may account for some of the striking differences in the estimated proportions of the genomes with low- and high recombination frequencies, our data do suggest that the recombination frequency in the honeybee is elevated across the entire genome, for regions of both low and high recombination alike. However, a more detailed analysis that directly compares homologous syntenic blocks in the genomes of *Apis* and *Nasonia* is necessary to substantiate this hypothesis.

### Association of Chromosome, Sequence, and Gene Parameters with the Local Recombination Rate

We studied 13 chromosome, sequence, and gene parameters in the parasitoid wasp genome and assessed their association with the recombination rate. A genome- and chromosome-wide screen for average values of these parameters (Table 1) suggested that a window–width of approximately 3.4 cM, corresponding with a physical window-width ranging from 73 Kb to 23 Mb (median 630 Kb), was appropriate to obtain accurate parameter estimates in each interval. The comparison of the 13 parameters with the recombination rate revealed several significant associations. The strongest association was found between the recombination rate and the physical distance from the center of the linkage group. While we have no data explicitly mapping the position of the chromosomes' centromeres, the fact that all of the *Nasonia* chromosomes are meta- or submetracentric strongly suggests that the centromeres map in or near the center of the inferred linkage groups. These regions show a very low recombination frequency (<1 cM/Mb) across all five chromosomes (Fig. 1). This finding is consistent with the well-known ‘centromere effect’ documented over a wide range of taxa, including the honeybee, and meaning a lack of recombination at the centromeres and in pericentromeric heterochromatin (e.g., [55]–[65]). We further found that the recombination rate in *Nasonia* is positively correlated with the GC content. The same association was reported by Beye et al. [21] for the honeybee and had previously been mentioned for other taxa as well (e.g., [62], [66]–[68]). Several lines of evidence suggest that the strong association of GC content and recombination could be due to a GC-biased repair of G∶T mismatches in heteroduplex DNA formed during recombination, particularly in groups of organisms like mammals which have a high level of methylated CpG which is hypermutagenic to TpG [69], [70]. Consistent with this idea is the recent finding that CpG dinucleotides in *Nasonia* undergo DNA methylation in a pattern that suggests its relevance for gene regulation [26]. Finally, we identified a significant positive association between the simple repeat content and the recombination rate. This association has also been documented across a wide range of taxa including the honeybee [21], [62], [71], [72]. However, the basis for this relationship is unclear. Some data indicate that simple repeats could be involved in the initiation of recombination [73]–[76], while others suggest that simple repeats are functionally involved in the regulation of gene expression [77]–[83]. The present data do not allow us to evaluate these two hypotheses.

The analysis of gene parameters in the parasitoid wasp genome revealed a strong positive correlation between the recombination rate and gene content. This contrasts with the data from the honeybee genome, where the two parameters apparently vary independently of each other [21]. A more detailed analysis of the *Nasonia* genome sequences showed that the strong association between the recombination rate and the gene content is due to a greater distance between genes and an increased size of their introns in regions of the genome with a low recombination rate, relative to those with a high rate. No significant correlation between the recombination rate and the size of introns has been found in the eusocial honeybee [21], and whether the recombination rate in the honeybee is correlated with the distance between genes is unclear: the reported *p* value of <0.014 is inconclusive when the significance level is adjusted for multiple testing.

Our data for the parasitoid wasp are in accordance with those from *Drosophila* and human [14], [16] where the recombination rate has a strong negative association with the size of introns. Comeron and Kreitman [16] proposed that a low recombination rate indirectly selects for larger introns, which reduce Hill-Robertson interference between adjacent exons and thus facilitate adaptive evolution. However, Prachumwat et al. [84] found that the recombination rate in *Caenorhabditis elegans* is positively associated with the size of introns and suggested that other (additional) factors influence intron size evolution. One such factor could be the gene expression level [17], [84], [85]. If recombination happens to occur more often in regions that contain highly expressed genes [86], and if highly expressed genes tend to have shorter introns [85], thereby reducing the cost of transcription, we would also expect to find an association between the recombination rate and the size of introns. Could this indicate that the observed correlation between the recombination rate and the size of introns in *Nasonia* may rather reflect an association between the recombination rate and gene expression? Currently, we cannot test this hypothesis as we have no genome-wide gene expression data available for *Nasonia*. The only clue indicating that differences in gene expression level are not the reason for the observed correlation between the recombination rate and the size of introns is that we do not see a comparable pattern when examining the size of exons, the number of exons per gene, and the overall size of proteins. If the transcription costs of highly expressed genes have been optimized, reducing the length of introns, we would expect a similar, although weaker, pattern for exons. A statistically significant dependency between gene expression and total exon length has indeed been found in *C. elegans*, but a similar analysis in humans failed to demonstrate this association [85]. However, differences in gene expression levels cannot explain the observed negative correlation between the recombination rate and the distance between genes. This correlation could be construed as a reduction of the Hill-Robertson effect between functional genomic elements in areas of low recombination and/or as a depletion of functional genomic elements in regions with a low recombination rate where more slightly deleterious mutations may accumulate [13].

Do we find evidence for an accumulation of slightly deleterious mutations in regions of the *Nasonia* genome with low recombination frequencies? Our data show an overall negative trend between the recombination rate and both the amino acid distance (*d*_A_) and the ratio of non-synonymous to synonymous substitutions per site (*ω*) when comparing *N. vitripennis* exon sequences with those of *N. giraulti* and *N. longicornis*. A negative correlation is expected if slightly deleterious mutations, and possibly also genes that are less constrained, accumulate in regions of the genome with low recombination frequency because of the reduced efficiency of natural selection. However, only the correlation between the recombination rate and *ω* when comparing *N. vitripennis* with *N. longicornis* proved to be statistically significant. Since males of *N. giraulti* tend to mate more frequently with their sisters than males of *N. longicornis* [87], [88], a smaller effective population size is expected for *N. giraulti*. One explanation for the lack of statistical significance could therefore be a lag in the removal of slightly deleterious non-synonymous mutations in this species [89], given the comparatively young age of the species group of 0.4–1.0 Myr [90]. In the honeybee, Beye et al. [21] also assessed the relationship between the recombination rate and the broad scale evolutionary rate. In contrast to the amino acid divergence and the ratio of non-synonymous to synonymous substitutions per site that we examined in the present investigation, they analyzed the proportion of most diverged genes by using the E value of the best hit when searching annotated honeybee genes against the Genbank non-redundant protein database. However, no significant association was found [21].

### Conclusions

The inference of the recombination frequencies and the analysis of chromosome, sequence, and gene parameters associated with it in the genome of the parasitoid wasp *Nasonia* confirmed that the honeybee genome differs in many features from the genomes of other taxa, even those belonging to the same order, the Hymenoptera. The genome-wide recombination rate in our parasitoid wasp is less than one tenth that of the honeybee, similar to the genome-wide recombination rate found in most other higher eukaryotes. Genes in regions of the *Nasonia* genome with a high local recombination rate have significantly smaller introns and are more densely packed than genes in regions with a low rate–a pattern that has also been found in most other organisms, but not the honeybee. In these respects, the genome of the haplodiploid wasp *Nasonia* resembles the genomes of diploid organisms, suggesting that the genomes of haplodiploid and diploid organisms do not have to differ systematically in their recombination rates and associated parameters. The exceptionally high recombination rate in eusocial Hymenoptera is most likely not therefore a consequence of haplodiploidy, but rather of some more honeybee-specific character, perhaps eusociality itself. With the availability of the *Nasonia* genome sequences and the high-density linkage map presented here, we have taken the first step in exploring the complex causes and consequences of the recombination rate differences between the honeybee and other taxa. However, the conspicuous discrepancy in the recombination frequencies between the two comparatively closely related taxa, *Apis* and *Nasonia*, is a striking phenomenon that still merits further investigation.

## Materials and Methods

### Mapping Population

Marker segregation data were collected from a population of 112, 12–16 h old male F_2_ hybrid embryos of *Nasonia vitripennis* (strain AsymCX, ♀) and *Nasonia giraulti* (strain RV2X(U), ♂). The same population had been used before by Niehuis et al. [33] to infer a *Nasonia* framework map with chromosomal anchored markers. The parental strains AsymCX and RV2X(U) are those for which the genome had been sequenced [26] and were generated in the Werren laboratory by inbreeding followed by antibiotic curing of the endosymbiont *Wolbachia* [91].

### Design of Locus and Allele Specific Oligonucleotides for SNP Genotyping

Using BLASTN [92] and applying the SEG filter for low complexity sequences, we searched for single nucleotide polymorphisms (SNPs) between NCBI REFSEQ predicted exons of the *N. vitripennis* genome and trace sequences of *N. giraulti*. We only considered SNPs that were i) flanked by 100 nucleotides (nt) up and downstream that were identical between *N. vitripennis* and *N. giraulti* and ii) verified by at least two identical *N. giraulti* trace sequences, since the *N. giraulti* trace sequence archive consists of unreplicated single-pass sequences. All *N. vitripennis* sequences with a SNP site were searched against the *N. vitripennis* genome and those with a second best BLAST hit with an E value<10^−10^ were discarded as non-unique sequences. A total of 2,293 sequence alignments were submitted to Illumina (Illumina Inc., San Diego, USA) for design of locus and allele specific oligonucleotide primers. For 2,035 of them, it was possible to design suitable primers for high-throughput SNP genotyping with the Illumina GoldenGate Genotyping Assay. Given the restricted capacity of the SNP genotyping microarray, however, only 644 were necessary to maximize the number of scaffolds represented on the array (Table S9).

We searched for extra SNPs in exon sequences of nuclear encoded genes of the oxidative phosphorylation (OXPHOS) pathway for a separate study [93] by allowing a narrower alignment window-width up and downstream of a potential SNP. For all 125 sequence alignments that had been submitted to Illumina suitable oligonucleotides were designed (Table S10). Finally, we designed oligonucleotides for 765 more SNPs without restricting the SNP search to coding sequences as described by Werren et al. [26] (Table S11).

### Microsatellite Markers

In addition to the 1,534 SNP markers for the Illumina GoldenGate Genotyping Assay and the 38 SNP, length polymorphic, and present/absent markers that had been genotyped in the mapping population by Niehuis et al. [33], we included 73 microsatellite markers from a study that will be published elsewhere (Leo Beukeboom, unpublished; data available upon request; Table S3).

### Molecular Procedures

Genomic DNA of the 112 male F_2_ hybrid embryos was isolated earlier by Niehuis et al. [33] and had been pre-amplified with the GenomiPhi DNA Amplification Kit (Amersham Biosciences, Piscataway, NJ) to cope with the small amount of extracted DNA (see [33]). We re-amplified aliquots of the DNA by using the same amplification kit and purified the DNA with a standard ethanol precipitation protocol (Note: previous work in our lab had shown that genotype data of pre-amplified and re-amplified genomic DNA from *Nasonia* embryos are fully consistent). We estimated the amount of amplified DNA with a NanoDrop 1000 spectrophotometer (NanoDrop Technologies, Wilmington, DE) and diluted the DNA of all samples with TE buffer (pH 8.0) to a final concentration of 50 ng per µl.

For SNP genotyping, we followed the Illumina Goldengate Genotyping Assay protocol provided by the manufacturer (Illumina Inc., San Diego, USA). All samples were hybridized to an Illumina Sentrix Array Matrix platform at The Translational Genomics Research Institute (Phoenix, AZ, USA), which was then scanned with an Illumina Beadstation 500 reader. The genotyping data were analyzed with the Illumina Beadstudio software.

Microsatellite markers were genotyped by separating their PCR products on a denaturing polyacrylamide gel as described by Niehuis et al. [33], [94] or on a 4% agarose gel. The PCR reaction set up for MS marker ‘mtRNApoly’ and the MS markers of the ‘Scaf’ series (Table S3) followed the protocol provided by Niehuis et al. [33], [94]. The applied PCR temperature profile started with a 5 min denaturation step at 95°C followed by 30 cycles of 1 min at 95°C, 1 min at 55°C, and 1 min at 72°C, followed by 10 min at 72°C. PCR amplification of the MS markers of the Nv100 and Nv300 series (Table S3) followed a protocol that will by published elsewhere (data available upon request).

### Linkage Map Reconstruction

Prior to linkage map reconstruction, we removed all markers with ≥10% missing information from the data set. We further excluded markers that showed one of the two parental genotypes at a frequency ≥70%; prior screening of a representative subset of the studied markers in the mapping population revealed neither of the two parental genotypes at a frequency higher than 59% [33]. The segregation data of the remaining markers were studied with the program MultiPoint ([95]–[97]; http://www.multiqtl.com). The optimal overall marker order was inferred following the protocol described by Niehuis et al. [33]. We subsequently visually checked the genotype data of each marker for plausibility and removed markers that differed in a genotype from that of immediately adjacent markers on both sides, since we regarded their deviating genotype(s) as highly improbable (see discussion). Map distances were calculated from the recombination fractions using Haldane's mapping function [98] and are given in centimorgans (cM).

### Recombination Rate Estimates

The genome-wide recombination rate was assessed by dividing the estimated genetic length of the linkage map (in cM) by i) the estimated physical size of the total *Nasonia* genome (312 Mb; [45]) and ii) the total size of the assembled *N. vitripennis* genome (295.1 Mb; [26]).

To approximate local recombination rates, we first estimated the genetic and the physical length of each marker cluster (i.e., group of markers in which the markers did not recombine) on the linkage map. The genetic length of a marker cluster was regarded as half the sum of its genetic distances to the two adjacent marker clusters (see also Table S7). The physical length of a marker cluster was estimated by summation of the lengths of the scaffolds or scaffold regions with markers on it that mapped in the marker cluster (see also Table S7 and Table S12). In cases in which a scaffold spanned more than one marker cluster, the physical length of the fraction of the scaffold in the marker cluster under consideration was estimated by i) calculating the distance between the two most distant markers on the scaffold in the specific marker cluster and ii) adding half of the distance(s) between the two nearest markers on the scaffold in the marker cluster under consideration and the adjacent marker cluster(s). The distance between the last mapped marker on either side of a scaffold and the actual end of the scaffolds was added to the marker cluster that included the nearest mapped marker on this scaffold.

We inferred local recombination rates by dividing the sum of the genetic lengths of two consecutive marker clusters by the sum of the two marker clusters' physical length. We thereby ensured that at least one recombination event had occurred within a given window along a chromosome. To visualize differences in the local recombination rate along the chromosomes, we applied a sliding window approach. For the statistical analysis, however, recombination rates as well as chromosome, sequence, and gene parameters (see below) were estimated for consecutive non-overlapping windows, since a sliding window approach artificially increases spatial autocorrelation due to non-independence of the data within each window relative to its neighbors. We omitted the first and the last marker cluster on each linkage group to account for the fact that its genetic and physical size are not well defined. Four additional marker clusters (i.e., 1.61, 3.53, 4.41, and 5.51; see Figure 1) had to be discarded from the statistical analyses, since four of the five chromosomes had an uneven number of marker clusters on the linkage map.

### Chromosome, Sequence, and Gene Parameters

The distance (in megabases, Mb) from the physical center of the linkage group of the non-overlapping windows to the center of a window was calculated using the physical size estimates for the 264 marker clusters (see above). GC content was calculated with the aid of a Perl script. Simple repeats were analyzed with the program *Msatfinder* and applying the *regex* search engine [99]. We considered *monomers* with more than eleven repeats as well as *di*-, *tri*-, *tetra*-, *penta*-, and *hexamers* with more than five repeats (*i.e.*, microsatellites in the narrow sense [100]).

To statistically test for an association of the recombination rate with specific gene parameters, we analyzed the distribution and characteristics of 10,769 predicted *N. vitripennis* genes on the mapped scaffolds of the non-overlapping windows using a GLEAN consensus gene set for *N. vitripennis* with a total of 15,216 predicted genes kindly provided by Christine Elsik (A&M University, Austin, TX; GLEAN6). We estimated gene density by counting the number of predicted genes for a given window and dividing by the estimated physical size (in Mb) of the window (N_g_ = 10,769). In cases where only part of a gene was laying in a window, we added the fraction of the gene's total length (defined as the distance between start and stop codon) laying in the window under consideration to the window's gene count. The average distance (in kilobases, Kb) between genes in a window was calculated for scaffolds or scaffold fragments with two or more predicted genes (N_d_ = 10,291). The average size of genes (i.e., number of amino acids, aa) and the average number of exons of a gene in a window was inferred only from genes contained entirely within the window under consideration (N_s_ = 10,656). The average size of exons and the average size of introns were deduced from all exons and introns in a window except those, which span more than one window (N_e_ = 71,550; N_i_ = 60,822).

To identify orthologous sequences of predicted *N. vitripennis* exons in the *N. giraulti* genome as well as in the *N. longicornis* genome, we used each mapped *N. vitripennis* exon contained entirely in the non-overlapping windows as a query against the *N. giraulti* and *N. longicornis* trace sequence archives using BLASTN [92] and applying the SEG filter for low complexity sequences and retained only those hits with an E value<10^−20^. Given that the *N. giraulti* and *N. longicornis* trace sequence archives consist of unreplicated single-pass sequences, we subsequently searched the *N. giraulti* and *N. longicornis* trace sequence archives with the respective hit sequence; only those sequences or sequence sections were considered for which a second trace archive sequence (E value<10^−20^) confirmed its nucleotide sequence. We then searched each putative *N. giraulti* and *N. longicornis* orthologous exon sequence against the *N. vitripennis* genome. We considered sequences as orthologs if the coordinates of the hit with the highest BLAST score and an E value<10^−20^ fell within the bounds of the initial *N. vitripennis* exon query sequence. All exon sequences were finally trimmed so that they started with the reading frame 0 and were a multiple of three nucleotides long. *N. giraulti* and *N. longicornis* sequences that included an internal stop codon were discarded. We obtained a total of 18,081 ortholog exon sequences or sequence sections for *N. giraulti* and 12,986 for *N. longicornis*. They respectively cover 17.1% and 12.2% of the predicted coding sequence in the *N. vitripennis* genome that map within the non-overlapping windows and 13.1% and 9.3% of the total predicted coding sequence of *N. vitripennis*.

All orthologous amino acid sequences were aligned with CLUSTAL W (1.83) [101] employing the BLOSUM62 substitution matrix. The alignment of the corresponding coding sequences was deduced from the amino acid alignments. Amino acid divergence (*d*_A_) were calculated with the PHYLIP software package [102] and using the PAM substitution matrix [103]. The ratio of non-synonymous to synonymous substitutions (*ω*) was estimated with the maximum likelihood method proposed by Goldman and Yang [104] and implemented in the software package PAML 4 [105]. Based on a parameter sensitivity analysis, we applied a codon substitution model that accounted for transitional rate bias (κ estimated) and unequal codon frequencies (F61 matrix).

Average values of the above specified chromosome, sequence, and gene parameters were calculated using the data from the non-overlapping windows.

### Statistical Analyses

We applied Dutilleul's modified *t* test [29] to assess correlations between variables using the program PASSaGE 2.0 [106]. The test corrects for spatial autocorrelation, i.e., the fact that values of variables sampled spatially close to one another are not necessarily independent from each other. The test requires information about the distance between all pairs of marker clusters, which we calculated using the genetic distance estimates from the inferred linkage map. The genetic distance between marker clusters from separate chromosomes was treated as missing information. The distance values were sorted into 15 contrast sets, each with an approximately equal number of observations (97–105). All tests were performed using ranked data, since we could not assume normal distributions.

## Supporting Information

Table S1 Markers removed due to missing genotypes or skewed genotype frequencies. Markers removed because genotype information is missing for 10% or more individuals and/or because they show one of the two genotypes with 70% or higher frequency.
(0.55 MB XLS)

Table S2 Markers removed due to disturbance of marker order or improbable genotypes. Markers removed from the dataset because they disturbed marker order stability or showed improbable genotypes.
(0.15 MB XLS)

Table S3 Studied MS, SNP, length polymorphic, and present/absent markers.
(0.05 MB XLS)

Table S4 SNP markers that did not map on any of the five *Nasonia* chromosomes. SNP markers that did not map on any of the five *Nasonia* chromosomes.
(0.02 MB XLS)

Table S5 Final genotype dataset used to infer high-density linkage map.
(2.43 MB XLS)

Table S6 Observed number of recombination events per chromosome and distance between them. Observed number of recombination events per chromosome and distance between them.
(0.03 MB XLS)

Table S7 Size and recombination rate estimates for marker clusters. Size and recombination rate estimates for marker clusters.
(0.05 MB XLS)

Table S8 Estimates of chromosome, sequence, and gene parameters. Chromosome, sequence, and gene parameters estimated for non-overlapping windows of two consecutive marker clusters each.
(0.09 MB XLS)

Table S9 SNP markers of *Nasonia* REFSEQ exon sequences.
(0.16 MB XLS)

Table S10 SNP markers of *Nasonia* OXPHOS pathway exon sequences.
(0.05 MB XLS)

Table S11 SNP markers of coding and non-coding sequences (Desjardins et al. in prep.).
(0.19 MB XLS)

Table S12 Estimated sections of *N. vitripennis* scaffold sequences within marker clusters.
(0.06 MB XLS)
